# Curbing the epidemic on both sides of the Atlantic: a public health perspective

**DOI:** 10.7448/IAS.17.4.19476

**Published:** 2014-11-02

**Authors:** Kevin Fenton

**Affiliations:** Health and Wellbeing, Public Health England, London, UK

## Abstract

HIV/AIDS continues to place a devastating toll on individuals, families and communities globally, and western industrialized countries are by no means exempt. Today, there are more than 1 million Americans and 100,000 Britons living with HIV, with a disproportionate burden of new and prevalent HIV infections borne by gay, bisexual and other men who have sex with men (MSM), racial/ethnic minorities, migrants and persons who use drugs. Epidemic concentration in urban areas, especially among: population sub-groups with high prevalence of risk behaviours; the socio-economically marginalized; or those with poor access to services, has been well documented. Recent increases in HIV incidence in the rural south US, and in MSM in both countries, reflect the dynamic and evolving nature of these epidemics. New national HIV prevention strategies in both countries have refocused attention on these domestic epidemics, prioritizing HIV testing scaling up, linkage to quality care and tackling long-standing health inequalities. There are also significant differences between the two countries – in part a reflection of the different health and social care systems; historical approaches to the funding and coordination of HIV prevention; and underlying patterns of health inequalities and their social and structural determinants. In addition, the social–political acceptability of using the sexual health frame to guide more holistic and integrated approaches to HIV prevention efforts remains a key difference. This presentation will compare and contrast HIV prevention responses in the US and UK over the past decade, identifying opportunities for enhancing the prevention response in these and other western industrialized countries in the 21st century.

